# Acquired diaphragmatic eventration

**DOI:** 10.11604/pamj.2024.48.67.44003

**Published:** 2024-06-25

**Authors:** Ashwin Karnan

**Affiliations:** 1 Department of Respiratory Medicine, Jawaharlal Nehru Medical College, Datta Meghe Institute of Higher Education and Research, Sawangi (Meghe), Wardha, Maharashtra, India

**Keywords:** Diaphragm, phrenic nerve, dyspnea

## Image in medicine

A 65-year-old female presented with complaints of breathing difficulty for the past 6 months that increased in intensity over the past 2 days. The patient has no known comorbid conditions and gave a history of road traffic accident where she had trauma to the chest wall and was managed conservatively. Computed tomography (CT) thorax showed eventration of the right dome of diaphragm. Arterial blood gas analysis showed respiratory failure and the patient was managed with non-invasive ventilator support. Due to persisting symptoms, diaphragmatic plication was done, and the patient improved symptomatically and is currently under follow-up. Diaphragmatic eventration is the elevation of a part or entire side of the diaphragm due to nerve or muscle injury or muscle development. It may be congenital or acquired. It is a rare disease, commonly affecting the left hemidiaphragm, with a male predominance. The gold standard investigation includes fluoroscopy and dynamic magnetic resonance imaging (MRI) scans. Complications are respiratory failure, pneumonitis, pleural effusion and deep vein thrombosis. Conservative management includes oxygen supplementation, physiotherapy, continuous positive airway pressure (CPAP) support and pulmonary rehabilitation. When they fail, plication may be done via open thoracotomy, video-assisted thoracic surgery (VATS), laparoscopic or open surgery. The prognosis is usually good and most patients may not need any intervention.

**Figure 1:**
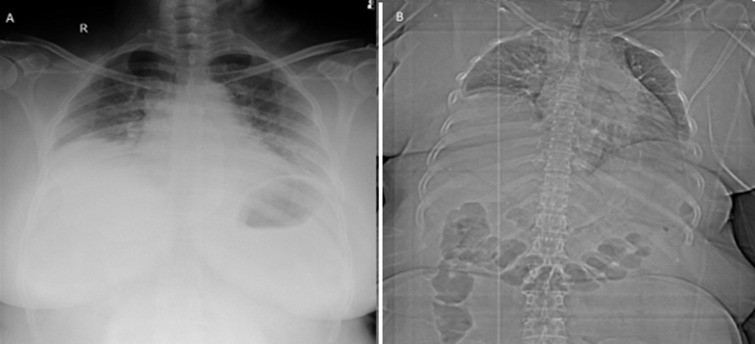
A) chest X-ray showing elevated right dome of diaphragm; B) computed tomography topogram of the patient showing eventration of right diaphragm

